# High Temperature Dry Tribological Behavior of Nb-Microalloyed Bearing Steel 100Cr6

**DOI:** 10.3390/ma14185216

**Published:** 2021-09-10

**Authors:** Yucheng Zhu, Jian Li, Chaolei Zhang, Wenjun Wang, Huan Wang

**Affiliations:** 1 School of Materials Science and Engineering, University of Science and Technology Beijing, Beijing 100083, China; b1820775@ustb.edu.cn; 2 School of Mechanical Engineering, Qinghai University, Xining 810016, China; s20180424@xs.ustb.edu.cn; 3 CITIC Metal Co. Ltd., Beijing 100004, China; s20200303@xs.ustb.edu.cn; 4 Science and Technology on Reactor Fuel and Materials Laboratory, Nuclear Power Institute of China, Chengdu 610213, China; weizitong@ustb.edu.cn

**Keywords:** bearing steel 100Cr6, niobium microalloying, high temperature dry tribological, wear volume, precipitation

## Abstract

100Cr6 steel is one of the most widely used bearing steels and a representative of first-generation bearing steel. Many engineering applications require rolling bearings to run at a high temperature. Therefore, it is necessary to improve the high temperature properties of 100Cr6 steel. In this paper, the effect of Nb on high temperature dry tribological behavior, including worn surface and friction coefficient, was analyzed by a wear test when Nb content was 0.018% and 0.040%. The results show that the microstructure is refined gradually, the hardness is improved, and wear volume decreases by 31.8% at most with the increase of Nb content. At 50 °C, the friction coefficient of 100Cr6 steel can be reduced by adding a small amount of Nb, but this effect will be weakened if the content of Nb is too high. In addition, excess Nb increases the hard precipitation of NbC, which aggravates the abrasive wear and leads to the increase in the depth of the worn surface. At 125 °C, the effect of Nb on tribological properties is weaker. With the increase of temperature, the steel substrate softens, and the oxide particles increase, which aggravates the abrasive wear and oxidation wear and makes the wear volume increase significantly.

## 1. Introduction

Although bearing steel has been developed for four generations, it is not a process of the new replacing the old, but a development process of gradually enriched varieties and gradual subdivision of the application field [1,2,3]. First-generation bearing steel is mainly composed of high carbon chromium bearing steel and is still the most widely used. Taking the world’s largest bearing producer as an example, China’s annual demand for bearing steel is about 3.7 million tons [4], and 100Cr6 and 100CrMn6 are still the main varieties, accounting for more than 95% of the total. During the bearing work, the existence of sliding friction inevitably causes wear of bearing parts [5]. If the wear resistance of the bearing steel is poor, the bearing will lose its accuracy prematurely, and the life of the bearing will decrease due to the decrease in rotation accuracy. The traditional bearing steel 100Cr6 has high hardness and good wear resistance, but it has defects in toughness and temperature bearing capacity. In recent years, many scholars have done a lot of research on the microstructure evolution and manufacturing process optimization of 100Cr6 steel [6,7]. Yusuke et al. [8] found that the quenched structure with almost no grain boundary carbides can be obtained by grain boundary amelioration (GBA) treatment, and the toughness can be increased to more than 5 times of the conventional one. Deep cryogenic treatment can improve the wear resistance and hardness of 100Cr6 bearing steel by promoting the conversion of the retained austenite into martensite [9]. However, the maximum ambient temperature can reach about 150 °C when the bearing steel works. Therefore, it is necessary to study the friction properties not only at room temperature, but also at high temperature, so as to improve the performance of traditional 100Cr6 steel.

At present, the most common way to improve wear resistance is to improve the hardness of materials [10,11]. The size of austenite grain plays a decisive role in improving the hardness of materials [12,13,14], and microalloying technology is usually used for grain refinement. Microalloy elements usually refer to niobium, vanadium, and titanium. The research of microalloying technology and the successful application of Nb steel production are mainly concentrated in the field of low carbon steel [15,16,17]. In recent years, many reports have shown that Nb in high carbon steel would refine grains by pinning at high temperature and dragging at low temperature to improve the microstructure and hardness [18,19,20]. Microalloying technology can also improve the thermal stability of high carbon steel [21]. Shi et al. improved the micro-hardness and wear resistance of the composite coating by preparing in situ NbC on the low carbon steel [22]. Our previous study has shown that a small amount of niobium would let the friction coefficient of bearing steel 100Cr6 decrease at room temperature [23]. The wear of bearings is not caused by a single mechanism. Abrasive wear and adhesive wear are the main wear mechanisms of the bearing [24]. Fatigue wear and oxidation wear occurred under some conditions [25]. A variety of mechanisms involved in the friction may lead to aggravation of wear. Microalloying will lead to the increase of precipitates, and the precipitates of alloying elements will affect the wear mechanism in the friction process [26,27]. Moreover, at high temperature, when the sliding friction speed is high, the oxidation will aggravate, and the oxide debris can flow or even melt [28,29]. It will lead to serious wear on the surface. Pan et al. established a new theory to understand the true coefficient of friction dependence on the hardness difference between parts of friction pairs, and verified pure hardness increase may not always be advantageous [30]. And Pei et al. verified that the change of temperature would affect the wear mechanism and matrix properties of the tested steel [31]. So far, the report on the high temperature friction properties of 100Cr6 steel is relatively limited. Therefore, the effect of Nb microalloying on high temperature tribological behavior of 100Cr6 steel is worthy of systematic investigation.

In this paper, the effects of niobium on high temperature dry tribological behavior of bearing steel 100Cr6 were studied. The worn surface, wear volume, and friction coefficient were studied, thereby providing some reference for the application of niobium microalloying technology and improvement of high temperature properties of 100Cr6 steel.

## 2. Materials and Methods

The materials used in this study are one Nb-free steel and two Nb-microalloyed steels. Apart from Nb content, the other alloy compositions of the steel are basically the same, and the specific alloy composition is shown in Table 1. The steelmaking, hot rolling and spheroidizing annealing processing for all three samples have been the same. After spheroidizing annealing, three samples were heat treated as follows: austenitizing at 850 °C for 1 mm/1.5 min, then oil quenching to room temperature, and tempering at 150 °C for 2 h according to the Chinese national standard GB/T 18254-2002.

### 2.1. Tribological Test

**Figure 1:**
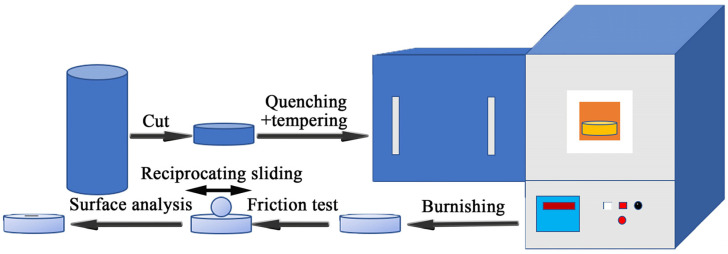
Schematic process flow chart showing the experimental procedures.

Samples of 7.88 mm in height and 24 mm in diameter were cut from the steel bar with a diameter of 28 mm, and both sides of the samples were mechanically polished. The final roughness of samples is about 300 nm. Before the experiment, the samples were cleaned with alcohol. Dry sliding wear tests were carried out under reciprocating sliding conditions at 50 °C and 125 °C. The contact load was 15 N corresponding to the initial Hertzian contact pressure [32] of 1.10 GPa, the frequency was 10 Hz, the stroke was 3 mm, and the wear time of each sample was 30 min. An optimal SRV-4 high temperature tribotester (Optimol, Munich, Germany) was used for the wear test. The size of the bottom specimen was φ24 mm × 7.88 mm. The final hardness of Nb-free steel, 0.018%Nb steel and 0.040%Nb steel are 61 HRC, 62.5 HRC and 62.5 HRC, respectively. The top specimen is a 100Cr6 steel ball with a diameter of 10.3 mm after induction quenching treatment, and the hardness was 65 HRC. The experimental process is shown in Figure 1.

### 2.2. Micro-Structural Analysis and Hardness Evaluation

An automatic inclusion system (EVO18) was used to automatically scan and count the number of precipitates >1 μm in the 5 mm × 5 mm field of view. The surface morphology and the depth of the worn surface was analyzed by an Olympus LEXT OLS4000 3D laser confocal microscope (Olympus, Tokyo, Japan). The microstructure of the steel was observed by a JSM-6480LV scanning electron microscope (SEM) (JEOL, Tokyo, Japan), and the worn surface morphology was observed at higher magnification. The surface hardness of the specimens was measured by a LEICA VMHT30M micro-Vickers hardness tester (Leica, Wetzlar, Germany).

## 3. Results and Discussion

### 3.1. Effect of Niobium on Microstructure

**Figure 2:**
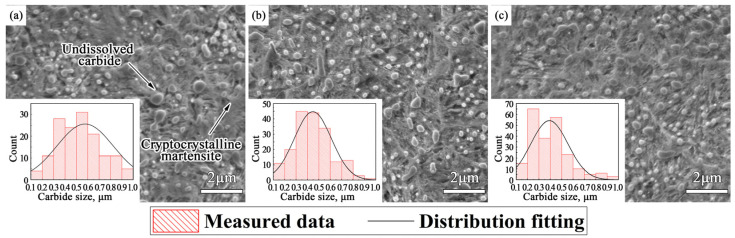
Scanning electron microscope micrographs of metallographic organization: (**a**) Nb-free steel, (**b**) 0.018%Nb steel, (**c**) 0.040%Nb steel.

Scanning electron microscope micrographs of the three experimental steels are shown in Figure 2. Microstructures of the samples were cryptocrystalline martensite and spherical undissolved carbides. The diameter distribution of spherical carbides in the 22.5 μm × 30.0 μm field of view was measured by nano-measurer software and the chord length method. The average sizes of carbide particles in Nb-free steel, 0.018%Nb steel and 0.040%Nb steel are about 0.58 μm, 0.45 μm, and 0.39 μm. Nb microalloying reduces the average diameter of undissolved carbides. Fine and uniformly distributed spherical carbides participate in the friction process as wear particles, which are helpful to enhance the wear resistance of bearing steel. In addition, the martensite content of the sample increased with the niobium content, because the niobium could increase the martensite start (Ms) point and promote the martensitic transformation [33].

### 3.2. Tribological Behavior

**Figure 3:**
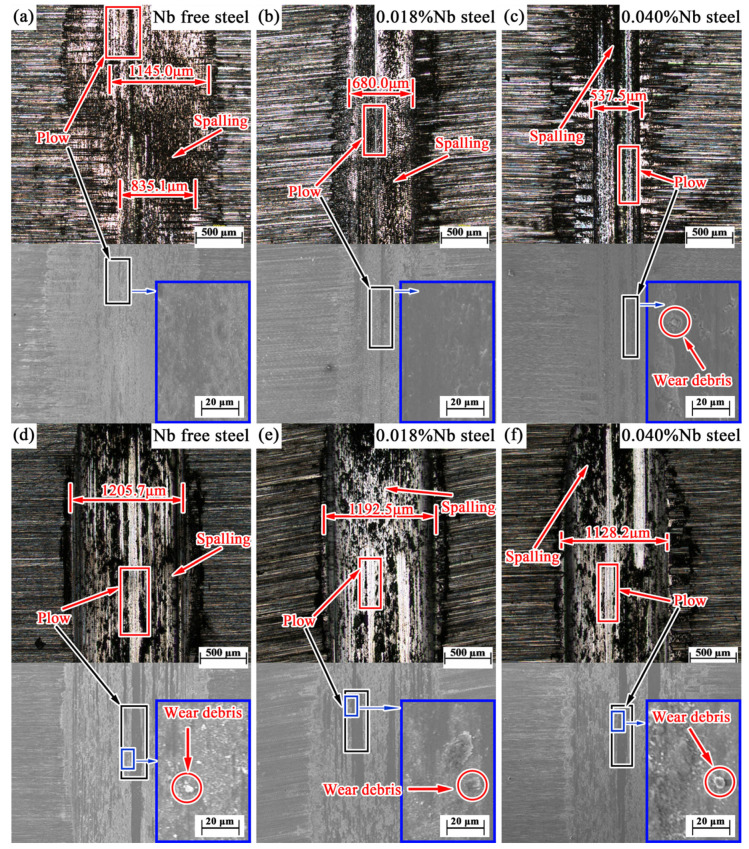
Worn surface morphology at (**a**–**c**) 50 °C and (**d**–**f**) 125 °C (optical micrographs above and SEM micrographs below).

Under the experimental conditions of 50 °C and 125 °C, the worn surface morphology of the specimen is shown in Figure 3. At 50 °C, the worn surface of the Nb-free steel is extremely uneven, and the width of the worn surface is inconsistent. The wide part reaches 1145.0 μm, and the narrow part is only 835.1 μm. The widths of the worn surface in the low Nb steel and high Nb steel are uniform, and compared with the Nb-free steel, the widths of the worn surface are significantly reduced, which are 680.0 μm and 537.5 μm, respectively. There are short furrows on the worn surface of Nb-free steel, while the furrows of low-Nb steel and high-Nb steel are longer and clearer. In the sliding friction process, material transfer and spalling occurred between the two contact surfaces. Under the action of reciprocating alternating contact stress, the continuous reciprocating sliding friction movement caused fatigue spalling of the worn surface. Adhesive wear and fatigue wear produced a certain number of abrasive particles, which led to abrasive wear. Therefore, the wear mechanisms of the three samples were mainly adhesive wear, fatigue wear, and abrasive wear of different degrees. At 125 °C, the average worn surface widths of the three steels are 1205.7 μm, 1192.5 μm, and 1128.2 μm, respectively. In addition to long and continuous furrows, there are many small and short furrows in Nb-free steel, and the furrows in 0.018%Nb steel and 0.040%Nb steel are mainly long and thick. The results show that the wear mechanism changed when the temperature went up from 50 °C to 125 °C. At 125 °C, the effect of Nb on tribological properties is weaker than that of temperature.

**Figure 4:**
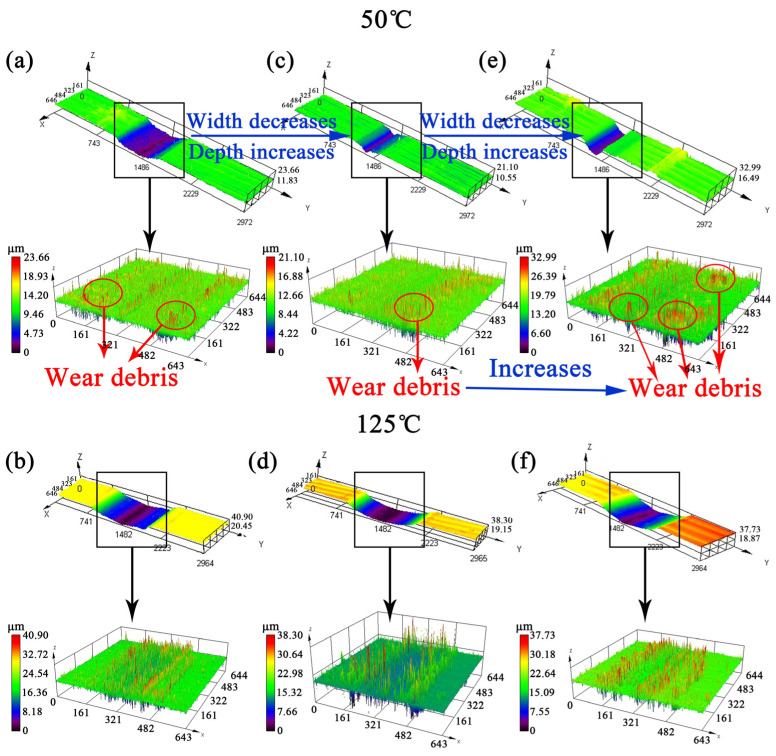
Three-dimensional morphology of worn surface: (**a**,**b**) Nb-free steel, (**c**,**d**) 0.018%Nb steel, (**e**,**f**) 0.040%Nb steel.

**Figure 5:**
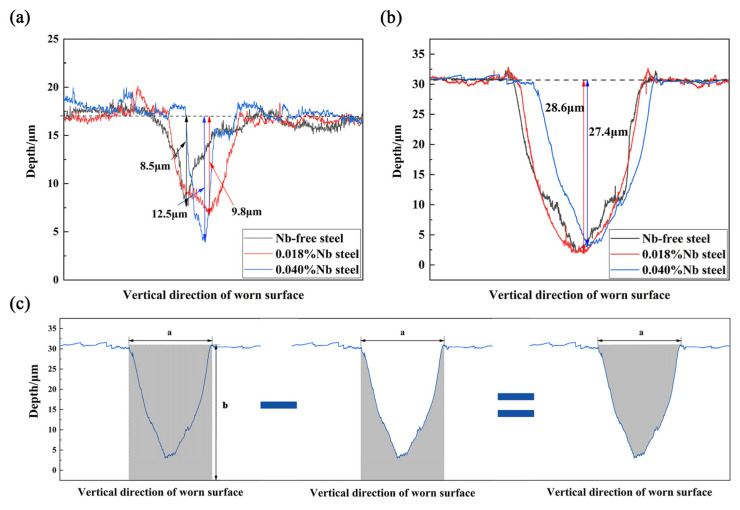
Undulation of worn surface at (**a**) 50 °C and (**b**) 125 °C, (**c**) calculation process of worn surface (a is the width of the worn scar, b is the height of the section perpendicular to the worn surface).

Three-dimensional morphology of the worn surface is shown in Figure 4. At 50 °C, the width of the worn surface decreases significantly, and depth gradually increases with the addition of Nb. But at 125 °C, the change of the width and depth of the worn surface is not obvious. Further observation of the interior of the worn surface shows that the sharp peaks in the figure represented wear debris. At 50 °C, 0.018%Nb steel had the least wear debris. The worn surface of 0.018% Nb steel was flatter than that of Nb-free steel and 0.040%Nb steel. At 125 °C, there were many abrasive particles on the worn surfaces of the three samples. Further analysis of the depth of the worn surface is shown in Figure 5. The result shows that the maximum depth of the worn surface in 0.040%Nb steel is 12.5 μm, while that in Nb-free steel and 0.018%Nb steel is smaller at 50 °C. At 125 °C, the maximum depth of worn surface in Nb-free steel and 0.018%Nb steel is 28.6 μm, and the maximum depth of worn surface in 0.040%Nb steel is 27.4 μm; there is no obvious difference in the three samples. If the shape of the worn surface is uniform in length direction, the length of the worn surface is 3 mm (the stroke in the experiment). The wear volume *V* is calculated according to Figure 5c and Equation (1):

(1) $$V=Z\times \left(A\times B-\int_{0}^{a}f\left(x\right)dx\right)$$

where *Z* (mm) is the length of the worn surface, *A* (mm) is the width of the worn surface, *B* (mm) is the *Y*-axis of the surface of the specimen not rubbed, and *f(x)* (mm) is a function of the depth of the worn surface. The results of the calculus show that the *V* of Nb-free steel, 0.018%Nb steel, and 0.040%Nb steel are 5.07 × 10^−3^ mm^3^, 4.06 × 10^−3^ mm^3^ and 3.46 × 10^−3^ mm^3^, respectively, at 50 °C. The wear volume decreased by 31.8% at most. The *V* of Nb-free steel, 0.018%Nb steel and 0.040%Nb steel are 24.48 × 10^−3^ mm^3^, 23.86 × 10^−3^ mm^3^, 21.42 × 10^−3^ mm^3^, respectively, at 125 °C. The wear volume decreased by 12.5% at most. Continuous high temperature in the friction process and wear effect inevitably led to the decrease of the tested steel substrate softening and strength. This increases the wear volume significantly when the temperature rises from 50 °C to 125 °C. This shows that the wear volume is reduced and the wear resistance is improved by adding Nb at these two temperatures, but this effect is weaker at 125 °C.

**Figure 6:**
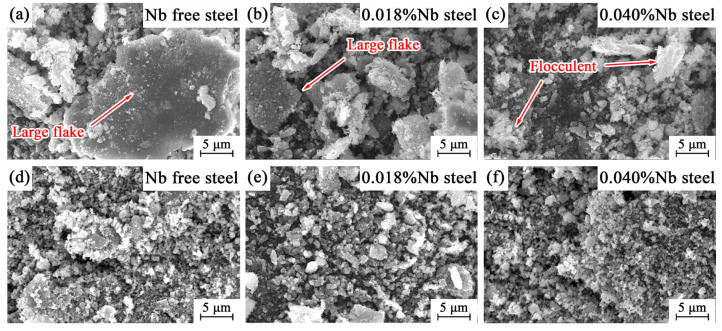
Wear debris morphology at different temperatures: (**a**–**c**) 50 °C and (**d**–**f**) 125 °C.

With the increase of the experimental temperature, the number of abrasive particles caused by adhesive wear and fatigue wear increased [34,35,36], and the oxidation rate increased significantly, and the amount of oxide debris increased sharply [37,38]. Some of the oxides fall off from the worn surface and are involved in the friction process, and another part of the oxides are crushed into smaller particles. These hard oxide particles will lead to abrasive wear in the exposed metal area, resulting in a large number of groove-like wear marks [39]. At 50 °C, wear debris of Nb-free steel and 0.018%Nb steel both have many large particles of peeling, as shown in Figure 6. The wear debris of 0.040%Nb steel was flocculent, and there were fewer large flakes. At 125 °C, the wear debris of the three samples was flocculent. The degree of oxidation increased with the increase of temperature. The oxide debris aggravates the abrasive wear of Nb-free steel and makes the depth of the worn surface of the three samples tend to be the same. This is one of the reasons why the wear volume of the three samples increased significantly at 125 °C. The wear mechanism mainly included adhesive wear, fatigue wear, abrasive wear, and oxidation wear at 125 °C.

**Figure 7:**
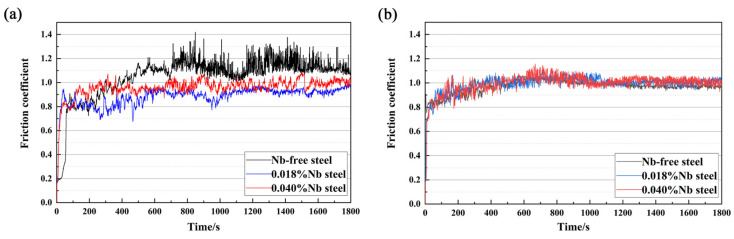
Curve of friction coefficient with time at different temperatures: (**a**) 50 °C; (**b**) 125 °C.

The friction coefficient mainly depends on the contact state between the friction surfaces [40]. The result of the experimental friction coefficient is shown in Figure 7. The average friction coefficient and error of the three samples were shown in Table 2. At the initial stage of wear, the friction coefficient of the three samples fluctuated greatly. With continuous wear time, the friction coefficient of the three samples gradually tended to become stable. When the experimental temperature was 50 °C, the friction coefficient of the three samples needed a long time to reach a stable state, and the fluctuation range of the friction coefficient of Nb-free steel was the largest, as shown in Figure 7a. The friction coefficient of 0.018%Nb steel was the lowest and its average value was 0.88. The friction coefficient of Nb-free steel was the largest, which was 1.05. The friction coefficient of 0.040%Nb steel was 0.96, which was lower than that of Nb-free steel, but higher than that of 0.018%Nb steel. However, at 125 °C, the friction coefficient of the three samples all reached a stable state in a short time, and the fluctuation range was small, as shown in Figure 7b. The friction coefficient of the three samples was essentially the same, which was about 1.00. The results show that the addition of Nb can decrease the friction coefficient of bearing steel at 50 °C. But excess Nb can weaken this effect. This is related to the increase in the number of abrasive particles on the worn surface.

### 3.3. Precipitates of Niobium Analysis

**Figure 8:**
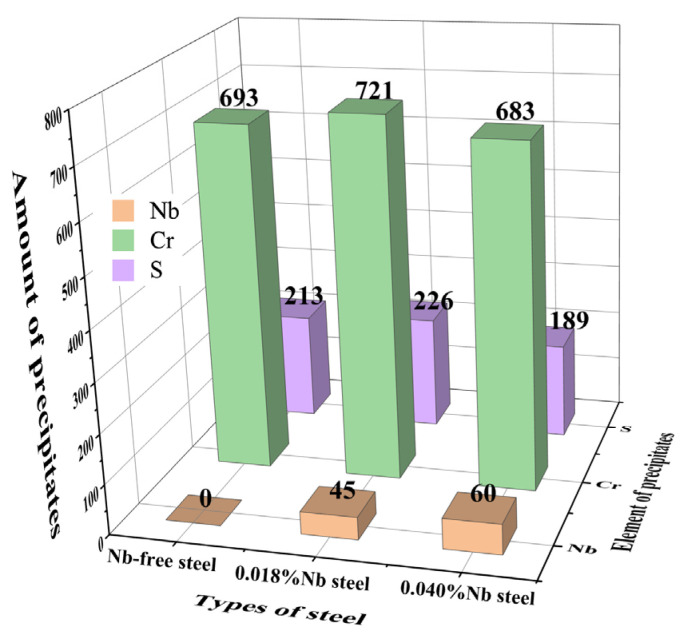
Statistics of precipitates in three steels.

The number of precipitates with a diameter of >1 μm in the 5 mm × 5 mm field of view in the three samples was automatically scanned and counted. The results are shown in Figure 8. It can be seen that there are a lot of carbide precipitates of Cr in the three samples due to the high carbon chromium bearing steel, and the difference in count is small. However, due to the difference in Nb content in the three samples, the number of Nb precipitates is different. There is no Nb added in Nb free steel, so the number of precipitates is 0, the 0.018%Nb steel is 45, and the 0.040%Nb steel is 60. From the analysis of the number of Nb precipitates, the amount of Nb precipitates increases slightly after adding 0.040%Nb.

**Figure 9:**
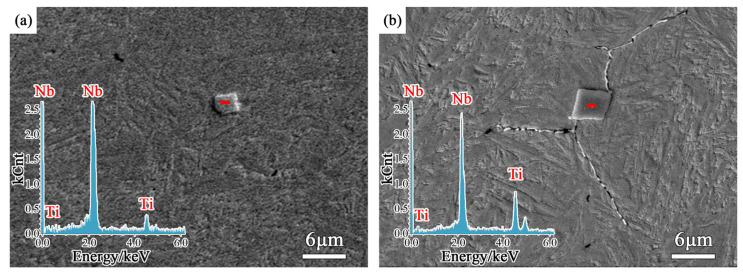
Micrograph of Nb-Ti carbide particles in Nb-microalloyed samples: (**a**) 0.018%Nb steel; (**b**) 0.040%Nb steel.

Carbides of 0.018%Nb steel and 0.040%Nb steel were analyzed by EDS, and the results are shown in Figure 9. Ti is a common harmful residual element in bearing steel, which is usually precipitated together with Nb. The higher the Ti content is, the sharper the edges and corners of the precipitates are. There were some Nb-Ti carbide precipitates in 0.018%Nb steel, which were block with 3~4 μm size. Some Nb-Ti carbide precipitates were observed in 0.040%Nb steel, which were blocked with 4~6 μm size. The hard precipitated phase NbC in the matrix is easier to wear as abrasive particles in the friction process. When the main wear mechanism changes from adhesive wear to abrasive wear, the width of the wear surface becomes narrower and the depth becomes deeper.

### 3.4. Effect of Niobium on Hardness

**Figure 10:**
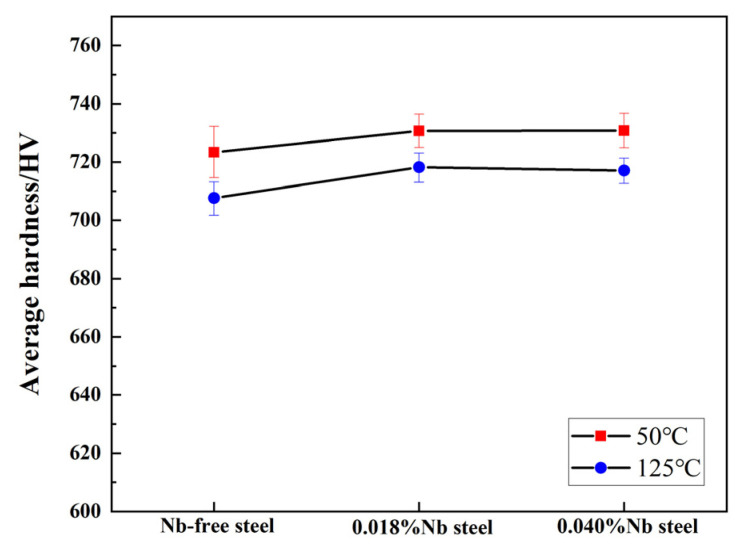
The hardness of non-friction surface after high temperature dry friction tests.

A related study has shown that in the dry friction test at room temperature, Nb mainly affects the friction performance by changing the hardness of the material [23]. The surface hardness of the specimen not rubbed after the friction test was measured, and the results are shown in Figure 10. After the experiment, the hardness of two types of Nb-microalloyed steels was higher than that of Nb-free steel at two temperatures. When the experimental temperature increases, the surface hardness of the specimen not rubbed after the friction test decreases, which proves that one of the reasons for the increase of wear volume is the softening of 100Cr6 steel substrate during the high temperature friction process. The hardness of Nb-free steel was the lowest, and less martensite content was the reason for low hardness. Hardness of 0.018%Nb steel was higher, because its martensite content was higher, too. Hardness of 0.018%Nb steel and 0.040%Nb steel were almost the same. The results show that Nb can still affect the friction properties by changing the hardness at high temperature.

## 4. Conclusions

The high temperature dry friction properties of high carbon bearing steel 100Cr6 with different Nb contents were studied, and the major conclusions drawn from the present investigation are listed as follows:

1. The addition of Nb affects the friction properties by changing the hardness. On the one hand, the addition of niobium refines the microstructure, increases the surface hardness, and improves the wear resistance. On the other hand, the addition of Nb makes the precipitated NbC hard particles become abrasive particles. At 50 °C, with the increase of Nb content from 0.018% to 0.040%, the increase of stray abrasive particles on the worn surface was the main reason leading to the increase of the average friction coefficient.
2. From 50 °C to 125 °C, the wear mechanism of the tested steel changed. At 50 °C, the wear mechanism was mainly adhesive wear, fatigue wear, and abrasive wear of different degrees. The increase in the number of NbC hard particles aggravated abrasive wear. At 125 °C, the wear mechanism mainly included adhesive wear, fatigue wear, abrasive wear, and oxidation wear. The high temperature can accelerate the formation of oxide wear debris, and then oxide debris can aggravate the abrasive wear of Nb-free steel and make the width and depth of the worn surface of the three samples tend to be the same.
3. The addition of Nb can decrease wear volume. The wear volume decreased by 31.8% at most at 50 °C. And this effect weakens with the increase of temperature. At 50 °C, the addition of 0.018%Nb can significantly reduce the wear loss and friction coefficient, and the depth of the worn surface is basically unchanged. At 125 °C, the effect of Nb on tribological properties is weaker. Considering all kinds of effects of Nb, in order to improve the high temperature friction and wear properties of 100Cr6 bearing steel, it is suggested to add a small amount of Nb.

## Figures and Tables

**Table 1:** Chemical composition of experiment steel 100Cr6 (wt.%).

Steel	C	Si	Mn	P	S	Cr	Nb	N
Nb-free steel	1.000	0.240	0.360	0.008	0.002	1.500	/	0.004
0.018%Nb steel	1.000	0.280	0.350	0.010	0.002	1.540	0.018	0.004
0.040%Nb steel	1.000	0.320	0.390	0.014	0.003	1.520	0.040	0.004

**Table 2:** Friction coefficient at two temperatures.

	50 °C		125 °C	
	Average	Standard Error	Average	Standard Error
Nb-free steel	1.05	0.02	1.01	0.01
0.018%Nb steel	0.88	0.01	0.98	0.01
0.040%Nb steel	0.96	0.02	1.00	0.02
